# Supplementary material for: Learning curve and surgical outcome of robotic assisted colorectal surgery with ERAS program
Source: Sci Rep. 2022 Nov 29;12:20566. doi: 10.1038/s41598-022-24665-w (PMC9709162; doi:10.1038/s41598-022-24665-w)
Supplement: Supplementary file 2 — Supplementary Information 2. [file 41598_2022_24665_MOESM2_ESM.zip › RAL Raw data-Ñ[▒K/105/Patho_ñw╝╨░O▒Kñσ.pdf]

臺中榮民總醫院　臺中市臺灣大道四段1650號　病理部活體組織切片　臨床醫學實驗及病理診斷中心 檢驗(查)報告

[redacted]

Pathologic diagnosis:
1. Colon, and terminal ileum, robotic right hemicolectomy --- Status post appendectomy with chronic xanthomatous inflammation and foreign body type giant cell reaction at pericecal soft tissue. The ileocecal valve, bilateral cut ends and mesocolic lymph nodes (0/19) are free of lesion.
2. Ileocolic root, excision --- Unremarkable lymph node (0/1).

Ancillary study for diagnosis:
IHC stains for AE1/AE3 (no abnormal epithelial cells) done on section F,G and H.

Gross description:
The specimen consists of 1) a segment of colon, 8 cm in length, and 8 cm in proximal circumference, 6.5 cm in distal cut end circumference, and a segment of terminal ileum, 6.5 cm in length, and 4 cm in cut end circumference. A pouch-like lesion, 3x2.5x1.3 cm, is noted, at the cecum. The ileocecal valve, and both cut ends are free of lesion. 2) a piece of tan soft tissue, 3.5x2x0.5 cm, labeled as "ileocolic root". All for section: A) specimen 2; Representative part of section: B) proximal cut end C) distal cut end D) ileocecal valve E+F,G-L) lesion at cecum all for section M-P) lymph nodes.
#T-59420_2 #M-43000_2 0 1105 000000
#T-C4560_2 #M-09183_2 0 1105 000000

已電子簽章

敬告：本單僅供醫師參考使用，不具任何申請效力！！
請病患妥為保管，若不慎遺失需自負個人資料外洩責任！
